# Clinicopathological characteristics of duodenal epithelial neoplasms: Focus on tumors with a gastric mucin phenotype (pyloric gland-type tumors)

**DOI:** 10.1371/journal.pone.0174985

**Published:** 2017-04-04

**Authors:** Takehiro Mitsuishi, Shigeharu Hamatani, Shinichi Hirooka, Nei Fukasawa, Daisuke Aizawa, Yuko Hara, Akira Dobashi, Kenichi Goda, Takahiro Fukuda, Masayuki Saruta, Mitsuyoshi Urashima, Masahiro Ikegami

**Affiliations:** 1 Department of Pathology, The Jikei University School of Medicine, Minato-ku, Tokyo, Japan; 2 Department of Endoscopy, The Jikei University School of Medicine, Minato-ku, Tokyo, Japan; 3 Division of Neuropathology, Department of Pathology, The Jikei University School of Medicine, Minato-ku, Tokyo, Japan; 4 Division of Gastroenterology and Hepatology, Department of Internal medicine, The Jikei University School of Medicine, Minato-ku, Tokyo, Japan; 5 Division of Molecular Epidemiology, Research Center for Medical Sciences, The Jikei University School of Medicine, Minato-ku, Tokyo, Japan; Shiga University of Medical science, JAPAN

## Abstract

**Objective:**

Epithelial tumors less commonly occur in the duodenum than in the stomach or large intestine. The clinicopathological characteristics of duodenal epithelial tumors remain a matter of debate. We therefore studied resected specimens to investigate the clinicopathological characteristics of duodenal epithelial tumors.

**Materials and methods:**

Among duodenal epithelial tumors resected endoscopically or surgically in our hospital, we studied the clinicopathological characteristics of 110 adenomas or intramucosal carcinomas. The grade of atypia of all tumors was classified into 3 groups according to the World Health Organization (WHO) 2010 classification. The tumors were immunohistochemically evaluated to determine the frequency of differentiation toward fundic glands.

**Results:**

As for patient characteristics, there were 76 men (75.2%) and 25 women (24.8%), with a median age of 65 years (range, 34 to 84). The tumors most commonly arose in the first to second part of the duodenum. Many lesions were flat, and the median tumor diameter was 8.0 mm. The lesions were classified into 2 types according to mucin phenotype: intestinal-type tumors (98 lesions, 89.1%) and gastric-type tumors (12 lesions, 10.9%). Intestinal-type tumors were subdivided into 2 groups: tubular-type tumors (91 lesions, 82.7%) and tubulovillous-type tumors (7 lesions, 6.4%). Gastric-type tumors were classified into 2 types: foveolar type (3 lesions, 2.7%) and pyloric gland-type (PG) tumors (9 lesions, 8.2%). The grade of atypia was significantly higher in gastric-type tumors (p<0.01). PG tumors were gastric-type tumors characterized by pyloric glands and findings suggesting differentiation toward fundic glands.

**Conclusions:**

About 10% of the duodenal tumors had a gastric-type mucin phenotype. Gastric-type tumors showed high-grade atypia. In particular, PG tumors showed similarities to PG tumors of the stomach, such as differentiation toward fundic glands.

## Introduction

Epithelial tumors less commonly occur in the duodenum than in the stomach or large intestine. Recent progress in endoscopic technology has increased the detection rate of epithelial tumors arising in the duodenum [1–3]. To our knowledge, however, large studies of resected specimens are scant, and the clinicopathological characteristics of duodenal epithelial tumors remain a matter of debate.

Tumors differentiating into Brunner’s glands have been reported in the duodenum [4–9]. Recently, many of these tumors have been found to be special tumors resembling pyloric gland-type (PG) tumors arising in the stomach. These tumors are characterized by the presence of a gastric mucin phenotype in superficial regions and hyperplasia of eosinophilic or clear mucous glands resembling pyloric glands in deep regions [10]. Some studies have reported that the presence of epithelial tumors with gastric features is associated with generative cell zones formed in Brunner’s glands during the process of mucosal regeneration [11–13]. Recently, duodenal tumors, including advanced cancer, have been studied [14], but the early stage of tumorigenesis limited to intramucosal lesions remains poorly understood.

We therefore studied resected specimens of 110 adenomas and intramucosal carcinomas to investigate the clinicopathological characteristics of duodenal epithelial tumors thought to be relatively early stage. We focused on the characteristics of PG tumors.

## Materials and methods

We studied adenomas or early carcinomas without invasion, selected from among duodenal epithelial tumors that were resected surgically or endoscopically in our hospital from January 1993 through June 2014. Only intramucosal lesions showing clear evidence of tumor atypia were studied. However, tumors arising in the papilla of Vater were excluded, in order not to include the tumors derived from a bile duct or pancreas. We studied 110 duodenal epithelial tumors from 101 patients that met the aforementioned conditions.

After confirming the age and sex of each patient and the location and macroscopic appearance of each tumor, all lesions were fixed in 10% formalin, longitudinally sliced into 2- to 3-mm segments, and embedded in paraffin. The paraffin-embedded specimens were then thinly sliced into 3- to 4-μm sections. The sections were stained with hematoxylin and eosin. Macroscopic appearance was classified as protruding (type I), flat (type IIa or IIb), or depression (type IIc or III). Maximum diameter was measured on the preparation as the tumor size. Immunohistochemical analyses were performed using primary antibodies against MUC5AC (CLH2; 1:50, Novocastra Laboratories, Newcastle upon Tyne, UK), MUC6 (CLH5; 1:50, Novocastra Laboratories), CD10 (56C6; 1:50, Novocastra Laboratories), MUC2 (Ccp58; 1:100, Novocastra Laboratories), Pepsinogen-1 (8003 [99/12]; 1:200, AbD Serotec, Kidlington, UK), and H,K-ATPase (1H9; 1:2000, MBL, Nagoya, Japan). Positive staining for pepsinogen-1 or H,K-ATPase in regions accounting for at least 5% of the tumor was defined as significant positive staining. Diaminobenzidine was used as a chromogen.

Among these antibodies, MUC5AC is a marker for gastric foveolar epithelial cells, and MUC6 is a marker for the gastric pyloric glands, gastric mucous neck cells (subsidiary cells), and Brunner’s glands in the duodenum. CD10 is a marker for the brush border of intestinal epithelial cells. MUC2 is a marker for goblet cells. Pepsinogen-1 is a marker for gastric chief cells and mucous neck cells. H,K-ATPase is a marker for parietal cells in the stomach.

**Fig 1:**
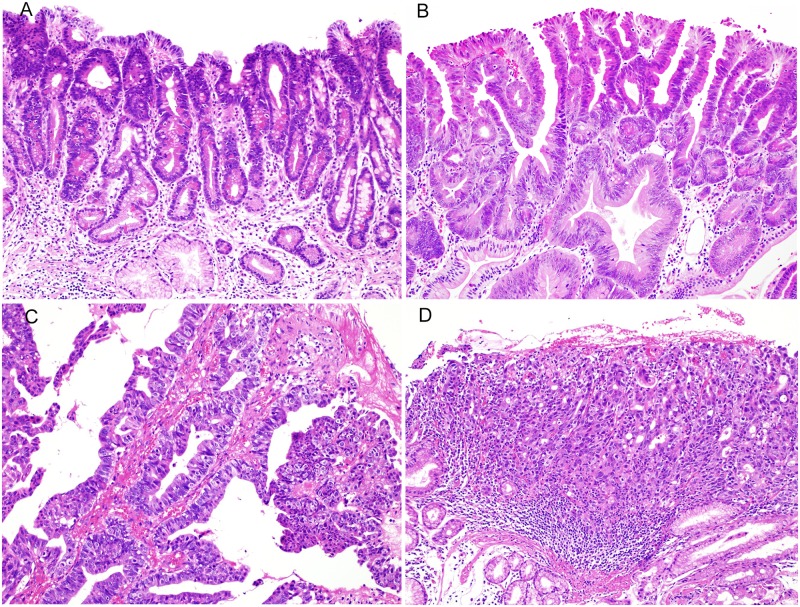
The grade of atypia of gastric tumors according to the World Health Organization (WHO) 2010 classification. (A) Low-grade intraepithelial neoplasia (HE staining). The tumor ducts consisted of epithelial cells with mild to moderate nuclear atypia. Stratified nuclei and mitotic figures were inconspicuous. (B) High-grade intraepithelial neoplasia (HE staining). The tumor ducts consisted of epithelial cells with high-grade nuclear atypia associated with stratified nuclei and mitotic figures. (C) Intramucosal carcinoma (HE staining). The formation of irregular branching of small ducts and cribriform ducts was evident, with distinct invasion to the stroma of the lamina propria. (D) Intramucosal carcinoma (HE staining). Tumor cells with severe structural atypia show solid and cord-like proliferation, associated with distinct invasion to the stroma of the lamina propria.

The grade of atypia of all tumors was estimated in the most highly atypical region and was classified into 3 groups according to the World Health Organization (WHO) 2010 classification [15]: low-grade intraepithelial neoplasia (LGIN), high-grade intraepithelial neoplasia (HGIN), and intramucosal carcinoma (IC). LGIN is characterized by the presence of glandular epithelial cells with mild to moderate nuclear atypia, the arrangement of nuclei in the glandular base, and no conspicuous stratified nuclei (Fig 1A). HGIN is characterized by the presence of severe nuclear atypia, conspicuous mitotic figures, stratified nuclei, and the protrusion of nuclei into the lumen (Fig 1B). IC is characterized by the presence of remarkable nuclear atypia and polar disturbance, distinct structural atypia such as abnormal branching in tumor glands or the presence of cribriform glands, and lesions invading the stroma of the lamina propria (Fig 1C and 1D).

The histological findings of tumors and the grade of atypia were assessed by three pathologists, who evaluated all of the lesions. For lesions for which the diagnosis differed, the diagnosis made by two of the three pathologists was adopted.

In addition, non-tumor mucosa in the study lesions was also stained with pepsinogen-1 and H,K-ATPase to examine the frequency of fundic glands in the normal mucosa of the duodenum.

Chi-square tests were used to compare the grades of atypia between intestinal-type tumors and gastric-type tumors and the frequencies of differentiation into fundic glands between pyloric-gland tumors and other tumors.

This study was conducted from April 2014 through May 2015. Because this retrospective study used paraffin-embedded specimens of duodenal tumors resected in routine medical treatment, informed written consent was not obtained from the individual patients. We disclosed the information about our protocol, and provided patients with the opportunity to refuse participation in our study. Our study was conducted in accordance with ethical guidelines for medical research in human subjects and was approved by the Ethics Committee of the Jikei University School of Medicine for Biomedical Research, Registration Number: 25–345 (7480).

## Results

### Patient characteristics and lesion characteristics

Table 1 and S1 Table shows the clinical and histopathological characteristics of the patients and lesions in our study. There were 110 tumors from 101 patients. Three patients had multiple (7, 2, or 3) tumors. Among the 110 tumors (101 cases), 5 tumors (5 cases) were resected surgically and 105 tumors (96 cases) were resected endoscopically (90 lesions [81 cases] by endoscopic mucosal resection [EMR], and 15 lesions [15 cases] by endoscopic submucosal dissection [ESD]).

**Table 1:** Clinicopathological characteristics of lesions.

**Number of lesions**		110
**[Number of patients]**		[101]
**Sex**	Male	76 (75.2%)
	Female	25 (24.8%)
**Median age (range)**		65 (34–84) years
**Median tumor diameter (range)**		8.0 (1–50) mm
**Tumor site in duodenum**	1: Superior part	22 (20.0%)
	2: Descending part	71 (64.5%)
	3: Horizontal part	11 (10.0%)
	4: Ascending part	6 (5.5%)
**Macroscopic appearance**	Protruding	30 (27.3%)
	Flat	65 (59.1%)
	Depressed	15 (13.6%)
**Histological atypia**	LGIN	44 (40.0%)
	HGIN	59 (53.6%)
	IC	7 (6.4%)

LGIN: Low-grade intraepithelial neoplasia, HGIN: High-grade intraepithelial neoplasia, IC: Intramucosal carcinoma

As for patient characteristics, there were 76 men (75.2%) and 25 women (24.8%), with a median age of 65 years (range, 34 to 84). The median tumor diameter was 8.0 mm (range, 1 to 50). As for the macroscopic appearance, 30 lesions (27.3%) protruded, 65 (59.1%) were flat, and 15 (13.6%) were depressed. As for the histological atypia, 44 lesions (40.0%) were LGIN, 59 lesions (53.6%) were HGIN, and 7 lesions (6.4%) were IC. The median diameter of 7 lesions diagnosed to be IC was only 8.0 mm (range, 5 to 50).

### Classification of duodenal epithelial tumors

**Fig 2:**
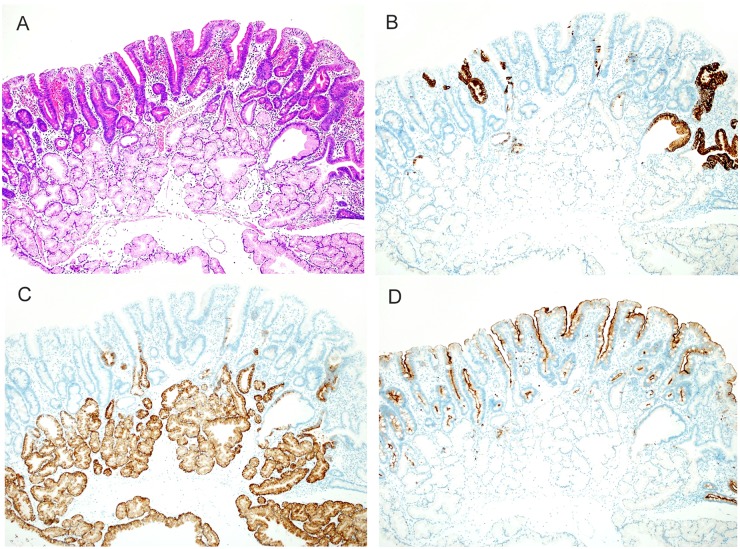
Histologic images of tubular type belonging to intestinal-type. (A) A hematoxylin and eosin (HE)-stained image. Many tumors were flat and had a tubular structure. (B) Mucin phenotype of tumor (MUC5AC). Among 91 lesions, scattered MUC5AC-positive cells were seen in 70 lesions (76.9%). (C) Mucin phenotype of tumor (MUC6). Same lesion as that shown above in B. Some tumor cells were positive for MUC6. Positivity for MUC6 was also seen in Brunner’s glands. (D) Immunohistochemical findings of tumor (CD10). Most tumor cells, excluding MUC5AC- and MUC6-positive cells, were positive for CD10.

**Fig 3:**
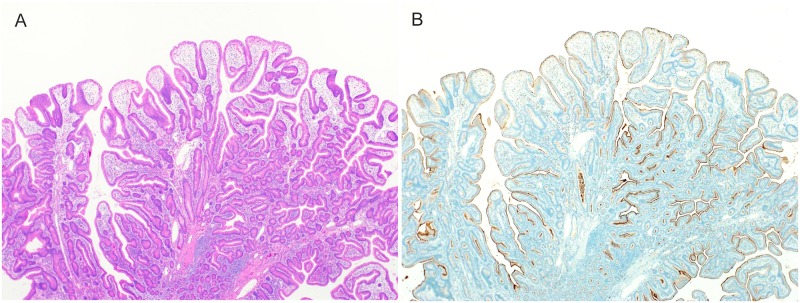
Histologic features of tubulovillous type belonging to intestinal-type. (A) An HE-stained image, showing intermingling of villous and tubular structures, forming a protruding lesion. (B) Immunohistochemical findings of tumors (CD10), showing that all tumor cells were positive for CD10.

**Fig 4:**
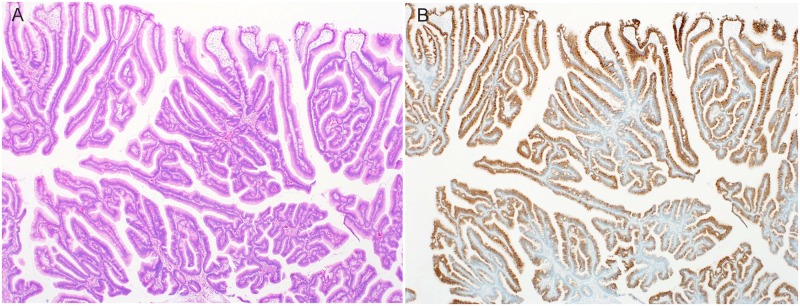
Histologic images of foveolar type belonging to gastric-type. (A) An HE-stained image, showing a protruding lesion consisting of gastric foveolar epithelial cells. (B) Mucin phenotype of tumor (MUC5AC). All tumor cells were positive for MUC5AC.

**Fig 5:**
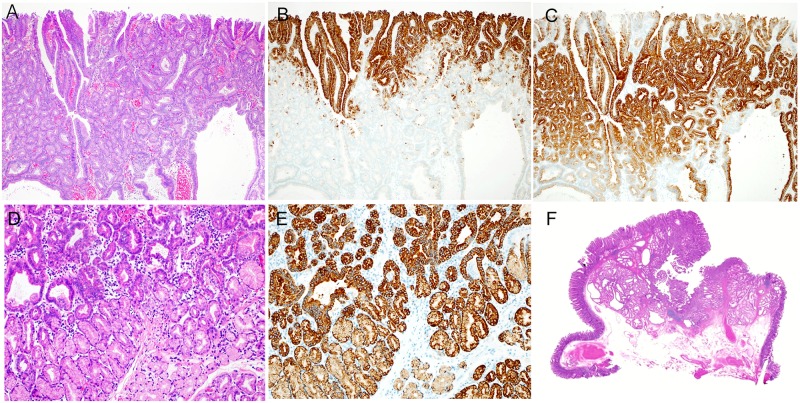
Histologic characteristics of Pyloric Gland-type (PG) belonging to gastric-type. (A) The surface layer of a tumor (HE staining), showing hyperplasia of small atypical ducts and dilated atypical ducts. (B) The surface layer of a tumor (MUC5AC antibody staining), showing the presence of MUC5AC-positive cells in the superficial layer of the tumor. (C) The surface layer of a tumor (MUC6 antibody staining), showing the presence of MUC6-positive cells in a large area, excluding the superficial layer of the tumor. (D) The deep region of a tumor (HE staining), showing hyperplasia of small atypical ducts. A region showing hyperplasia of clear cells with no atypia was found in the deepest part of the tumor. (E) A deep part of a tumor (MUC6 antibody staining), showing the presence of MUC6-positive cells in a large area extending from the atypical ducts to the region of clear-cell hyperplasia. (F) A magnified image of a tumor (HE staining), showing a distinctly protruding lesion. A region of tumor duct and mucous duct hyperplasia was found in the lamina propria. The muscularis mucosa was compressed and pushed down deeply.

Duodenal epithelial tumors were classified into 2 groups according to their mucin phenotype: 98 (89.1%) intestinal-type tumors and 12 (10.9%) gastric-type tumors. Tumors with CD10-positive brush borders of the intestinal epithelium were classified as intestinal-type tumors. MUC2 staining was used to label goblet cells, but the results were difficult to evaluate owing to large differences in the amounts of goblet cells among the intestinal-type tumors. Intestinal-type tumors were subdivided into 2 groups: 91 (82.7%) tubular-type tumors, which had small tubular glands composed of absorptive epithelium, and 7 (6.4%) tubulovillous-type tumors, which had a villous structure composed of absorptive epithelium. On the other hand, tumors that were composed nearly entirely of epithelium with MUC5AC-positive gastric mucin phenotype were classified as gastric-type tumors. Gastric-type tumors were classified into 2 types: 3 (2.7%) foveolar-type tumors, with a phenotype resembling that of the gastric foveolar epithelium, and 9 (8.2%) pyloric gland-type (PG) tumors in the duodenum, with a phenotype resembling that of gastric pyloric glands (Figs 2–5). Table 2 and S1 Table show the clinicopathological characteristics of each type of lesion.

**Table 2:** Clinicopathological characteristics of tumors according to histologic type.

		Intestinal type		Gastric type	
		Tubular type	Tubulovillous type	Foveolar type	Pyloric-gland type
**Number of lesions**		91 (82.7%)	7 (6.4%)	3 (2.7%)	9 (8.2%)
**Median tumor size(mm)**		7.5 (1–50)	19.1 (8–50)	9.7 (8–12)	15.3 (11–28)
**Location**	1	14 (15.4%)	2 (28.6%)	3 (100%)	3 (33.3%)
	2	62 (68.1%)	4 (57.1%)	0 (0.0%)	5 (55.6%)
	3	9 (9.9%)	1 (14.3%)	0 (0.0%)	1 (11.1%)
	4	6 (5.5%)	0 (0.0%)	0 (0.0%)	0 (0.0%)
**Macroscopic appearance**	Protruding	14 (13.1%)	6 (85.7%)	3 (100%)	8 (88.9%)
	Flat	62 (67.4%)	1 (14.3%)	0 (0.0%)	1 (11.1%)
	Depressed	15 (16.9%)	0 (0.0%)	0 (0.0%)	0 (0.0%)
**Histological atypia**	LGIN	40 (44.0%)	4 (57.1%)	0 (0.0%)	0 (0.0%)
	HGIN	45 (49.5%)	3 (42.9%)	3 (100%)*	8 (88.9%)*
	IC	6 (6.5%)	0 (0.0%)	0 (0.0%)	1 (11.1%)*

* p<0.01

LGIN: Low-grade intraepithelial neoplasia, HGIN: High-grade intraepithelial neoplasia, IC: Intramucosal carcinoma

### 1. Tubular type (Fig 2)

Histologically, tubular-type tumors in the duodenum were characterized by atypical tubular glands lined by eosinophilic absorptive epithelium. All tumor tubules were positive for CD10. Of these lesions, 70 (76.9%) showed focal intermingling of gastric-type mucin phenotypes positive for MUC5AC and for MUC6.

Ninety-one lesions (82.7%) were tubular type. Many lesions were small and flat, with a median tumor diameter of 7.5 mm (range, 1 to 50) and arose in the second portion of the duodenum. As for the grade of atypia, 40 lesions (44.0%) were LGIN, 45 (49.5%) were HGIN, and 6 (6.5%) were IC.

### 2. Tubulovillous type (Fig 3)

Histologically, tubulovillous-type tumors of the duodenum were characterized by the presence of eosinophilic cytoplasm, similar to tubular tumors, and the proliferation of atypical tubules with conspicuous villous structures. All cases consisted of only CD10-positive cells, with no gastric-type mucin phenotypes.

Seven lesions (6.4%) were tubulovillous type. The median tumor diameter was 19.1 mm (range, 8 to 50). All lesions but 1 were distinctly protruding type. As for the grade of atypia, 4 lesions (57.1%) were LGIN, and 3 (42.9%) were HGIN.

### 3. Foveolar type (Fig 4)

Histologically, foveolar-type tumors were characterized by tall columnar cells resembling gastric foveolar epithelium and a tubulovillous structure. All tumors were composed of MUC5AC-positive cells, with few MUC6-positive cells and no intermingling of intestinal-type mucin phenotype.

Three lesions (2.7%) were foveolar type. The median tumor diameter was 9.7 mm (range, 8 to 12), and the lesions were distinctly protruding type. As for the grade of atypia, all lesions were HGIN.

### 4. Pyloric Gland type (PG) (Fig 5)

PG tumors of the duodenum resembled PG tumors of the stomach and distinctly protruded into the intestinal lumen. Histologically, PG tumors were characterized by proliferation of small tubular ducts with severe atypia in the surface layer of the tumor and inverted proliferation towards the muscularis mucosa. Deep in the tumor the proliferation of slightly eosinophilic mucous glands with low-grade atypia could be seen. The surface layer of the tumor was immunohistochemically positive for MUC5AC. Positivity for MUC6 was extensively seen deep in the tumor.

Nine lesions (8.2%) were PG tumors. The median tumor diameter was 15.3 mm (range, 11 to 28). All lesions but 1 were distinctly protruding type. The grade of atypia was HGIN in 8 lesions (88.9%) and IC in 1 lesion (11.1%). All PG tumors had a severe atypical region, and no PG tumors were classified as LGIN.

### Comparison of grade of atypia between gastric type and intestinal type

The tumors were divided into 2 groups according to the grade of atypia. LGIN was defined as low-grade atypia, and HGIN and IC were defined as high-grade, severe atypia. The grade of atypia was then compared between intestinal-type tumors and gastric-type tumors. All 12 gastric-type tumors showed high-grade, severe atypia, and the difference was significant on analysis with a chi-square test (p<0.01).

### Pepsinogen-1 and H,K-ATPase immunohistochemistries of duodenal epithelial tumors and normal mucosa surrounding tumors

**Fig 6:**
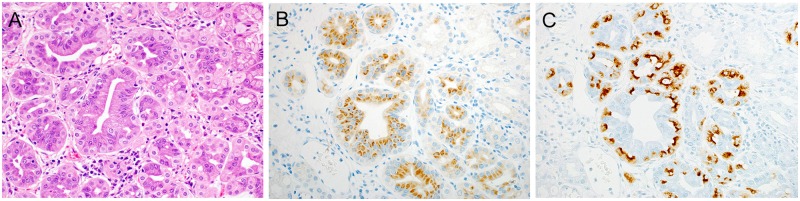
Differentiation of pyloric-gland type to fundic glands. (A) HE-stained image, showing proliferation of atypical glands associated with mildly enlarged nuclei. (B) Differentiation into fundic glands (pepsinogen-1). Tumor glands are positive for pepsinogen-1. (C) Differentiation into fundic glands (H,K-ATPase). Similar to pepsinogen-1, H,K-ATPase-positive cells can be seen in tumor glands.

The results of pepsinogen-1 and H,K-ATPase immunohistochemistries are listed in Table 3 and S1 Table. Pepsinogen-1 or H,K-ATPase immunoreactive cells were observed in the relatively low-grade atypical region of tubular glands of gastric-type tumors (Fig 6). An analysis of the presence or absence of pepsinogen-1 expression and H,K-ATPase expression in PG tumors and other tumors showed that the expression of both antibodies was significantly higher in PG tumors on analysis with a chi-square test (p<0.01).

**Table 3:** Immunohistochemical findings of tumors and fundic glands in normal mucosa.

			Intestinal type		Gastric type	
			Tubular type (n = 91)	Tubulovillous type (n = 7)	Foveolar type (n = 3)	Pyloric-gland type (n = 9)
**Neoplastic lesions**	Pepsinogen-1	+	0 (0%)	0 (0%)	0 (0%)	7 (77.8%)*
		−	91 (100%)	7 (100%)	3 (100%)	2 (22.2%)
	H,K-ATPase	+	0 (0%)	0 (0%)	1 (33.3%)	7 (77.8%)*
		−	91 (100%)	7 (100%)	2 (66.7%)	2 (22.2%)
**Non-neoplastic lesions (n = 110)**	Pepsinogen-1 or H,K-ATPase		16 (14.5%)			

* p<0.01

In 16 (14.5%) of 110 tumors, pepsinogen-1 or H,K-ATPase immunopositive cells were observed in the normal mucosa surrounding the tumors. The frequency was independent of the duodenal tumor type. Positive cells in normal mucosa were seen near the surface layer of Brunner’s glands.

### Patient prognosis

Tumors, regardless of the grade of atypia and mucin phenotypes, were completely resected in all cases, and no cases had recurrence or progression.

## Discussion

### Characteristics of patients and lesions

We therefore analyzed the clinicopathological characteristics of 110 adenomas and intramucosal carcinomas from among duodenal epithelial tumors that were resected surgically or endoscopically. Although epithelial tumors of the small intestine are rare, in recent years, cases resected endoscopically are increasing, and endoscopic techniques for diagnosis are improving [1–3]. We studied 110 duodenal epithelial intramucosal tumors resected completely from 101 patients.

Many of the patients were elderly men (76 men, 75.2%). The ratio of male patients was reported to be 63.6% [16] and 73.0% [17]. And, the most common macroscopic appearance of the tumors was flat type (65 lesions, 59.1%). Overall, 93 lesions (84.5%) arose in the first or second portion of the duodenum. The ratio of duodenal epithelial tumors arising in the proximal duodenum was reported to be 76.3% [14], 90.5% [16], and 92.6% [18]. These results are similar to our findings. However, care should be exercised before concluding that the most common site of duodenal epithelial tumors is the proximal portion of the duodenum, because it is difficult to examine the distal portion of the duodenum by upper gastrointestinal endoscopy [19]. In addition, the median tumor diameter was 8.0 mm in our study. However, the median diameter of 7 lesions diagnosed to be IC was only 8.0 mm (range, 5 to 50). In a report, atypia of depressed region of duodenal tumors is considered to be higher [20]. Therefore, caution is required because even relatively small tumors can be associated with high-grade atypia.

### Classification of duodenal epithelial tumors

In our study, 110 duodenal epithelial tumors were classified into 12 gastric-type tumors (10.9%) and 98 intestinal-type tumors (89.1%). Intestinal-type tumors were accounted for the majority of duodenal epithelial tumors. In the intestinal-type tumors, 44 lesions were LGIN, 48 lesions were HGIN, and 6 lesions were IC. Tubular-type tumors were most common (91 lesions, 82.7%). However, of these lesions, 70 (76.9%) showed focal intermingling of gastric-type mucin phenotypes positive for MUC5AC and for MUC6. During the process of mucosal healing, generative cell zones are formed in Brunner’s glands, and participate in gastric foveolar metaplasia and tumorigenesis [11–13,21]. We considered that this is the reason why many tubular-type tumors had focal gastric mucin. Only 7 lesions (6.4%) were tubulovillous type and had the largest diameter among the lesion types and distinctly protruded.

As for gastric type, all lesions were highly atypical lesions (HGIN or IC) and more lesions arose from the proximal duodenum than intestinal type. Gastric-type tumors developed in association with protrusion, and there is a case report of duodenal obstruction caused by a giant gastric-type tumor [22]. Only 3 lesions (2.7%) were foveolar type, distinctly protruded, and were relatively small, with a median diameter of 9.7 mm. All foveolar-type tumors were composed of MUC5AC-positive cells, so they may arise via the same mechanism as tubular type. In our study, however, the mechanism remained unclear.

### Pyloric gland-type tumors and fundic glands of the duodenum

PG tumors are well known in the stomach, but arise in the duodenum and gallbladder rarely [11,23–27]. In our study, 9 lesions (8.2%) were PG tumors. PG tumors showed differentiation into fundic gland cells frequently. We considered that cells capable of differentiating into fundic glands participated in the occurrence of PG tumors.

Among 110 specimens of normal mucosa included with the lesions studied, the presence of fundic glands was confirmed in 16 specimens (14.5%) (Table 3) and the frequency was independent of the duodenal tumor types. Regardless of the duodenal tumor types, fundic glands were present in the normal mucosa at a certain rate. The prevalence has been reported to range from 2% to 20% [28–30], which is generally consistent with the frequency in our study. Fundic glands in the duodenum were found near the surface layer of Brunner’s glands. In some previous studies, generative cell zones in the Brunner’s glands have been reported to arise and generate gastric foveolar metaplasia at the time of mucosal regeneration [11,12], and 12 of 15 cases of fundic glands in the duodenum were reported to be associated with peptic ulcer [31]. Namely, the occurrence of not only gastric foveolar metaplasia but also fundic glands may be associated with mucosal regeneration after inflammation. However, it may be difficult to accurately classify the morphologic characteristics of fundic glands as congenital or acquired in the duodenum.

And then, fundic gland cells were found in 77.8% of PG tumors, and PG tumors included higher proportions of fundic gland cells than other types of tumors (Table 3). Matsubara et al. reported that gastric foveolar metaplasia, fundic glands, and PG tumors in duodenum were associated with *GNAS* mutations [32,33]. On the basis of these findings and our results, we consider that the cells capable of differentiating into fundic glands might occasionally participate in the development of PG tumors.

PG tumors of the stomach and duodenum have been reported to have similar histologic characteristics and genetic features [33,34]. Namely, Kushima et al. reported that PG tumors arising in the stomach preferentially occurred in the regions of fundic glands and showed that these tumors differentiated into chief cells and mucous neck cells (auxiliary cells) in 8 of 12 patients [35]. In our study, PG tumors of the duodenum were also clearly found to differentiate into fundic glands, similar to PG tumors of the stomach. In molecular biological studies of gastric and duodenal PG tumors, *GNAS* mutations were found frequently, which indicates molecular biological similarities between PG tumors of the stomach and those of the duodenum [33,34].

As described above, recently, duodenal tumors, especially gastric-type tumors, have attached attention, and accumulation of more cases is important.

## Conclusions

Most lesions of duodenal epithelial tumors were classified as intestinal-type tumors. However, 10.9% of the tumors had a gastric-type mucin phenotype characterized by protruding growth and high-grade atypia.

Nine (8.2%) PG tumors of all duodenal tumors showed similarities to PG tumors of the stomach, such as differentiation into fundic glands. PG tumors are considered to arise from cells capable of differentiating into fundic glands by differentiation into pyloric gland-like cells.

## Supporting information

S1 Table Clinicopathological, histological and immunohistochemical findings of all tumors. (XLSX)

## References

[1] Endo M, Abiko Y, Oana S, Kudara N, Chiba T, Suzuki K, et al Usefulness of endoscopic treatment for duodenal adenoma. Dig Endosc. 2010; 22: 360–365. 10.1111/j.1443-1661.2010.01014.x 21175499

[2] Tsuji S, Doyama H, Tsuji K, Tsuyama S, Tominaga K, Yoshida N, et al Preoperative endoscopic diagnosis of superficial non-ampullary duodenal epithelial tumors, including magnifying endoscopy. World J Gastroenterol. 2015 11 7. 10.3748/wjg.v21.i41.11832 PMC4631981 26557007

[3] Maruoka D, Arai M, Ishigami H, Okimoto K, Matsumura T, Nakagawa T, et al Cold polypectomy for nonampullary duodenal adenoma. Endoscopy. 2015; 47 (Suppl 1). 10.1055/s-0034-1392668 26479293

[4] Dorandeu A, Raoul JL, Landemore G, Rioux-Leclercq N, Loget P, Taouji S, et al Adenocarcinoma of Brunner’s glands: an entity exceptionally described. Report of case. Ann Pathol. 1995; 15: 211–215. 7639859

[5] Itsuno M, Makiyama K, Omagari K, Tanaka T, Hara K, Tsuda N, et al Carcinoma of duodenal bulb arising from the Brunner’s glands in ulcer healing. Gastroenterol Jpn. 1993; 28: 118–125. 10.1007/BF02775012 8382639

[6] Kawamoto K, Motooka M, Hirata N, Masuda K, Ueyama T, Yasukouchi A, et al Early primary carcinoma of the duodenal bulb arising from Brunner’s glands. Gastrointest Endosc. 1994; 40: 233–236. 8013831 10.1016/s0016-5107(94)70176-8

[7] Miyamoto T, Matsuba S, Yokoyama Y, Ito M, Takeuchi T, Sugimura M, et al Early duodenal cancer supposedly arising from the Brunner’s gland, report of case. Stomach and Intestine. 1991; 26: 1395–1399.

[8] Zanetti G, Casadai G. Brunner’s gland hamartoma with incipient ductal malignancy. Report of a case. Tumori. 1981; 67: 75–78. 7245358 10.1177/030089168106700114

[9] Ajioka Y, Watanabe E, Narisawa R, Iwabuchi M, Kobayashi M, Maeo S, et al Primary Duodenal Tumors and Tumor-like Lesions, Focused on Their Incidence and Brunner’s Gland Adenoma. Stomach and Intestine. 1993; 28: 627–638.

[10] Khor TS, Brown I, Kattampallil J, Yusoff I, Kumarasinghe MP. Duodenal adenocarcinoma arising from a pyloric gland adenoma with a brief review of the literature. BMJ Case rep. 2010; 10.1136/bcr.10.2010.3385 PMC3028104 22802482

[11] Kushima R, Manabe R, Hattori T, Borchard F. Histogenesis of gastric foveolar metaplasia following duodenal ulcer: a definite reparative lineage of Brunner’s gland. Histopathology. 1999; 35: 38–43. 10383712 10.1046/j.1365-2559.1999.00681.x

[12] Kushima R, Stolte M, Dirks K, Vieth M, Okabe H, Borchard F, et al Gastric-type adenocarcinoma of the duodenal second portion histogenetically associated with hyperplasia and gastric-foveolar metaplasia of Brunner’s glands. Virchow Arch. 2002; 440: 655–659. 10.1007/s00428-002-0615-z 12070607

[13] Sakurai T, Sakashita H, Honjo G, Kasyu I, Manabe T. Gastric foveolar metaplasia with dysplastic changes Brunner gland hyperplasia: possible precursor lesions for Brunner gland adenocarcinoma. Am J Surg Pathol. 2005; 29: 1442–1448. 16224210 10.1097/01.pas.0000180449.15827.88

[14] Ushiku T, Arnason T, Fukuyama M, Lauwers GY. Extra-ampullary Duodenal Adenocarcinoma. Am J Surg Pathol. 2014; 38: 1484–1493. 10.1097/PAS.0000000000000278 25310836

[15] Lauwers GY. Gastric carcinoma In: Bosman FT, Carneiro F, Hruban RH, Theise ND, editors. WHO classification of Tumours of the Digestive system 4th Edition Lyon: IARC publications; 2010 pp. 55–56

[16] Terada T. Malignant tumors of the small intestine: A histopathologic study of 41 cases among 1,312 consecutive specimens of small intestine. Int J Clin Exp Pathol. 2012; 5: 203–209. 22558474 PMC3341676

[17] Kakushima N, Kanemoto H, Sasaki K, Kawata N, Tanaka M, Takizawa K, et al Endoscopic and biopsy diagnoses of superficial, nonampullary, duodenal adenocarcinomas. World J Gastroenterol. 2015 5 14. 10.3748/wjg.v21.i18.5560 PMC4427679 25987780

[18] You HS, Hong JW, Yun EY, Kim JJ, Lee JM, Lee SS, et al Primary Non-ampullary Duodenal Adenocarcinoma: A Single-center Experience for 15 Years. Korean J Gastroenterol. 2015; 66: 194–201. 10.4166/kjg.2015.66.4.194 26493504

[19] Bandi M, Scagliarini L, Anania G, Pedriali M, Resta G. Focus on the diagnostic problems of primary adenocarcinoma of the third and fourth portion of the duodenum. Case report. G Chir. 2015; 36: 183–186. 10.11138/gchir/2015.36.4.183 26712074 PMC4732589

[20] Tomizawa M, Shinozaki F, Motoyoshi Y, Sugiyama T, Yamamoto S, Ishige N. Duodenal Adenocarcinoma Diagnosed from a Biopsy Specimen of a Depressed Lesion Obtained by Magnifying Endoscopy. Case Rep Gastroenterol. 2016 5 4. 10.1159/000444441 PMC4929374 27403120

[21] Xue Y, Vanoli A, Balci S, Reid MM, Saka B, Bagci P, et al Non-ampullary-duodenal carcinomas: clinicopathologic analysis of 47 cases and comparison with ampullary and pancreatic adenocarcinomas. Mod Pathol. 2016 10 14. 10.1038/modpathol.2016.174 27739441

[22] Guan CS, Ma da Q, Hu HY, Liu WH. A Giant Gastric Adenoma Embedded in the Duodenum. J Coll Physicians Surg Pak. 2015 4. 25933448

[23] Kojima E, Tsukahara S. A case of early gallbladder carcinoma concomitant with hyperplastic polyp of pyloric gland metaplasia. Journal of Japan Biliary Association. 2006; 20: 635–641.

[24] Nakai H, Tanabe S, Koizumi W, Kida Y, Imaizumi H, Kida M, et al Endoscopic Diagnosis of Protruding Lesion with Gastric Covering Epithelium in the Duodenal Bulb. Stomach and Intestine. 2001; 36: 1499–1505.

[25] Kim K, Jang SJ, Song HJ, Yu E. Clinicopathologic Characteristics and Mucin Expression Brunner’s Gland Proliferating Lesions. Dig Dis Sci. 2013; 58: 194–201. 10.1007/s10620-012-2320-3 22836185

[26] Friedrich-Rust M, Jaeger C, Gossner L, May A, Günter E, Stolte M, et al Early Duodenal Adenocarcinoma Arising in Gastric Metaplasia Treated by Endoscopic Resection. Z Gastroenterol. 2006; 44: 323–328. 10.1055/s-2006-926492 16625461

[27] Takahashi M, Hamada S, Nakamura K, Ihara E, Iju M, Suda K, et al The Characteristics of Adenoma and Carcinoma Arising from Brunner’s Gland. Stomach and Intestine. 2011; 46: 1619–1625.

[28] Mann NS, Mann SK, Rachut E. Heterotopic gastric tissue in the duodenal bulb. J Clin Gastroenterol. 2000; 30: 303–306. 10777193 10.1097/00004836-200004000-00020

[29] Hoedemaeker PJ. Heterotopic Gastric Mucosa in the Duodenum. Digestion. 1970; 3: 165–173. 4915587 10.1159/000197027

[30] Johansen A, Hansen OH. Heterotopic gastric epithelium in the duodenum and its correlation to gastric disease and acid level. Acta Pathol Microbiol Scand A. 1973; 81: 676–680. 4771973 10.1111/j.1699-0463.1973.tb03560.x

[31] Wolff M. Heterotopic gastric epithelium in the rectum: a report of three new cases with a review of 87 cases of gastric heterotopia in the alimentary canal. Am J Clin Pathol. 1971; 55: 604–616. 5090217 10.1093/ajcp/55.5.604

[32] Matsubara A, Sekine S, Kushima R, Ogawa R, Taniguchi H, Tsuda H, et al Frequent GNAS and KRAS mutations in pyloric gland adenoma of the stomach and duodenum. J Pathol. 2013; 229: 579–587. 10.1002/path.4153 23208952

[33] Matsubara A, Ogawa R, Suzuki H, Oda I, Taniguchi H, Kanai Y, et al Activating GNAS and KRAS mutations in gastric foveolar metaplasia, gastric heterotopia, and adenocarcinoma of the duodenum. Br J Cancer. 2015 4 14. 10.1038/bjc.2015.104 PMC4402452 25867268

[34] Hida R, Yamamoto H, Hirahashi M, Kumagai R, Nishiyama K, Gi T, et al Duodenal Neoplasms of Gastric Phenotype: An Immunohistochemical and Genetic Study With a Practical Approach to the Classification. Am J Surg Pathol. 2016 12 14. 10.1097/PAS.0000000000000785 27984236

[35] Kushima R, Sekine S, Matsubara A, Taniguchi H, Ikegami M, Tsuda H. Gastric adenocarcinoma of the fundic gland type shares common genetic and phenotypic features with pyloric gland adenoma. Pathology International. 2013; 63: 318–325. 10.1111/pin.12070 23782334

